# Molecular Mechanisms of *Bartonella* and Mammalian Erythrocyte Interactions: A Review

**DOI:** 10.3389/fcimb.2018.00431

**Published:** 2018-12-12

**Authors:** Hongkuan Deng, Qiuxiang Pang, Bosheng Zhao, Muriel Vayssier-Taussat

**Affiliations:** ^1^School of Life Sciences, Shandong University of Technology, Zibo, China; ^2^UMR BIPAR, INRA, ANSES, École Nationale Vétérinaire d'Alfort, Université Paris-Est Créteil Val-de-Marne, Maisons-Alfort, France

**Keywords:** bartonellosis, erythrocyte interactions, adhesion and invasion, replicate and persist, pathogenesis factors

## Abstract

Bartonellosis is an infectious disease caused by *Bartonella* species that are distributed worldwide with animal and public health impact varying according to *Bartonella* species, infection phase, immunological characteristics, and geographical region. *Bartonella* is widely present in various mammals including cats, rodents, ruminants, and humans. At least 13 *Bartonella* species or subspecies are zoonotic. Each species has few reservoir animals in which it is often asymptomatic. *Bartonella* infection may lead to various clinical symptoms in humans. As described in the *B.tribocorum*-rat model, when *Bartonella* was seeded into the blood stream, they could escape immunity, adhered to and invaded host erythrocytes. They then replicated and persisted in the infected erythrocytes for several weeks. This review summarizes the current knowledge of how *Bartonella* prevent phagocytosis and complement activation, what pathogenesis factors are involved in erythrocyte adhesion and invasion, and how *Bartonella* could replicate and persist in mammalian erythrocytes. Current advances in research will help us to decipher molecular mechanisms of interactions between *Bartonella* and mammalian erythrocytes and may help in the development of biological strategies for the prevention and control of bartonellosis.

## Introduction

*Bartonella* species are fastidious, *Gram-negative* hemotropic organisms. *Bartonella* have been isolated from a range of species; from diverse animals, such as canids, rodents, ruminants, and felids. They are mainly transmitted via direct contact (animal scratches and bites) or by numerous arthropods such as sand flies, fleas, lice, biting flies, and ticks (Deng et al., 2012).

Until now, at least 40 species or subspecies of *Bartonella* have been found (Mullins et al., 2017). Each species can establish a lasting intraerythrocytic bacteraemia in its reservoir host, but typically not with obvious detriments (Vayssier-Taussat et al., 2009; Deng et al., 2012). In contrast, when *Bartonella* accidentally infects the incidental hosts, which means that absolutely no erythrocytes are involved during the acute phase of a zoonotic infection, the acute clinical manifestations can be provoked (Raoult, 2007; Mosepele et al., 2012).

*B. henselae* is the most prevalent zoonotic *Bartonella* species (Yuan et al., 2011). *B.henselae* infection is typically asymptomatic in the reservoir cats, in spite of up to 10^8^ CFU/ml blood. However, various clinical symptoms can be caused in humans, such as cat scratch disease and bacillary peliosis in immunocompetent and immunocompromised individuals, respectively (Dehio, 1999, 2001; Chomel et al., 2009; Pulliainen and Dehio, 2012; Deng et al., 2016). The distribution of *Bartonella* in animal and public health varies with *Bartonella* species, infection phase, immunological characteristics, and geographical region.

*Bartonella* spp., *Plasmodium* spp., *Babesia* spp., *Theileria* spp.*, Mycoplasma suis*, and *Anaplasma marginale* are important intracellular pathogens which can infect mammalian erythrocytes (Barbour and Restrepo, 2000; Schülein et al., 2001; Groebel et al., 2009). In contrast to other pathogens, all *Bartonella* species could survive within the infected erythrocytes for several weeks with only subtle changes of the erythrocyte membrane, except the deadly *B. bacilliformis* (Dehio, 2005; Harms and Dehio, 2012).

**Figure 1:**
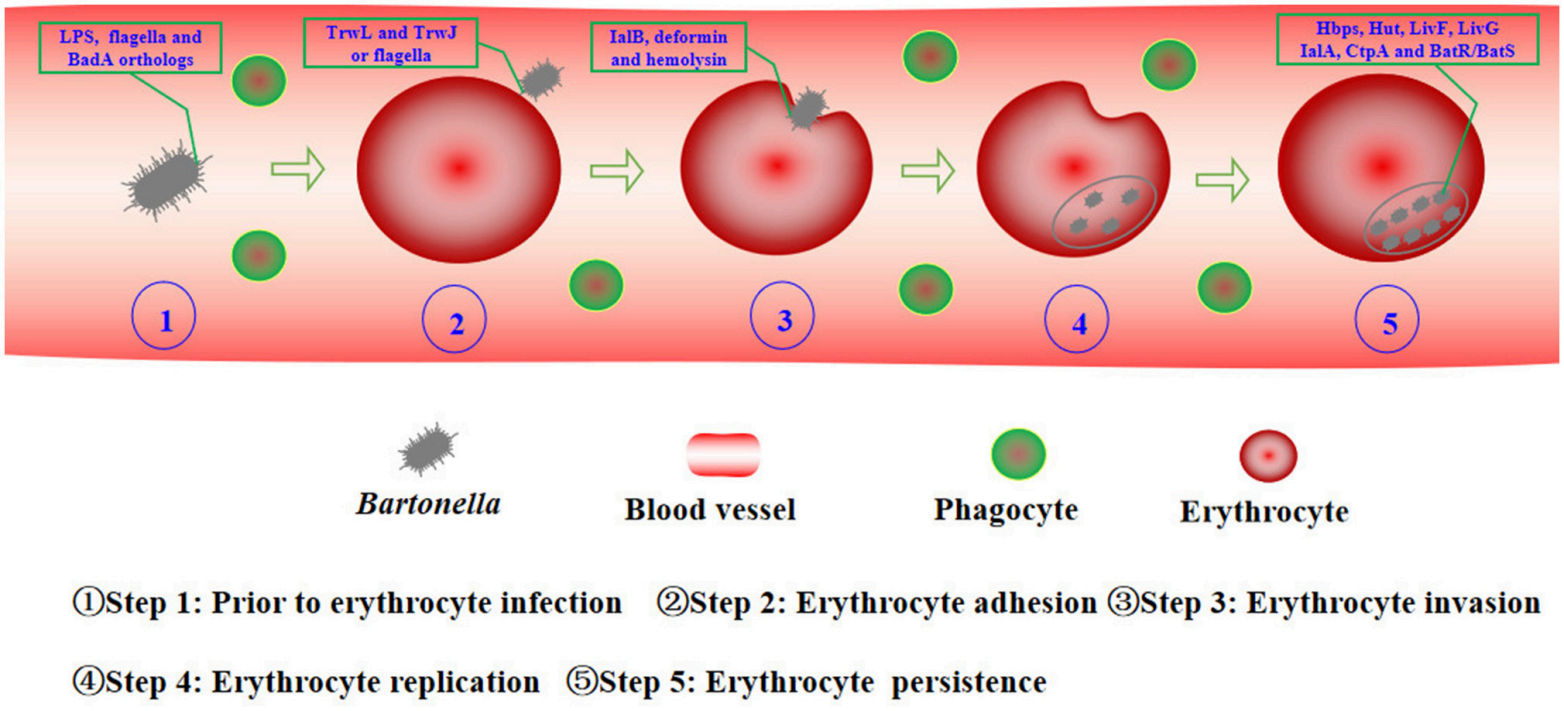
Holistic view of *Bartonella* interactions with erythrocytes. Prior to erythrocyte infection, *Bartonella* must escape the host immune responses to facilitate their extracellular longevity to approach and infect erythrocytes efficiently. *Bartonella* use LPS, flagella, and BadA orthologs against phagocytes and complement activation (Step 1). Erythrocyte adhesion is mediated by multiple copies of TrwL and TrwJ variants of pilus components or flagella (Step 2). IalB, deformin, and hemolysin cause some subtle changes of erythrocyte membrane and erythrocyte invasion by *Bartonella* species (Step 3). Once inside, the bacterium replicates in a membrane-bound compartment, until the number of intracellular bacteria reaches static levels for the remaining lifespan of the infected erythrocytes, the distinguishable changes will disappear (Step 4). Within erythrocytes, *Bartonella* uses Hbps, Hut, LivF, and LivG to get nutrients, and IalA, CtpA, and BatR/BatS to cope with stressors (Step 5).

The infection course of *Bartonella* has been described in natural and experimental animal models, such as the *B. birtlesii*-mouse, *B.tribocorum*-rat, and *B. henselae*-cat models (Guptill et al., 1997; Boulouis et al., 2001; Seubert et al., 2002; Birtles, 2005; Marignac et al., 2010). All of them show similar results, which suggest a universal infection course of the different species in their respective mammalian reservoir hosts. Following initial inoculation, *Bartonella* could be rapidly cleared from the blood, which was considered due to *Bartonella* infection of the so called primary niche outside of circulating blood, potentially endothelial cells, erythrocytic precursors, liver, and possibly other cell types or organs (Dehio et al., 1997; Dehio, 1999, 2001; Mändle et al., 2005; Deng et al., 2012b). *Bartonella* was released into the blood stream between 2 and 5 days post-infection. Followed by erythrocyte adhesion and invasion. They then replicated in the infected erythrocytes until eight daughter cells were reproduced. The infected erythrocytes could persist for many weeks (Schülein et al., 2001; Guptill, 2010; Harms and Dehio, 2012). This review will discuss the current understanding of *Bartonella* and erythrocyte interactions, especially focusing on the required factors involved in virulence of *Bartonella* in their reservoir hosts (Figure 1).

## Step 1: Prior to Erythrocyte Infection

As mentioned above, prior to mammalian erythrocytes infection, *Bartonella* could infect the primary niche and reappear in the bloodstream. *Bartonella* must escape the host immune responses to facilitate their extracellular survival to approach and infect erythrocytes efficiently in this step (Arvand et al., 2001; Kabeya et al., 2003; Resto-Ruiz et al., 2003; Ben-Tekaya et al., 2013; Dehio and Tsolis, 2017; Scherler et al., 2017).

### The First Strategy Is Replication of Large Numbers of *Bartonella*

Following intravenous inoculation, the bacteria were unable to infect the erythrocytes. Instead, they were disappeared from circulation and maintained undetectable during ~4 days (Schülein et al., 2001). During this time, the primary niche may support *Bartonella* replication and allow them to gain competency for erythrocyte interactions (Dehio, 2005; Harms and Dehio, 2012). On day 5 post-inoculation, numerous *Bartonella* are seeded into the bloodstream and cause autoagglutination (Kaiser et al., 2008; Schmidgen et al., 2014). This is one of the first steps of biofilm formation (Okaro et al., 2017; Tu et al., 2017). The bacterial factors that are responsible for replication are presently unknown.

### The Second Strategy Is Against Phagocytes

On day 5 post-inoculation, *Bartonella* are extracellular, thus they are completely exposed to the immune system. Phagocytes such as macrophages are the first line of immune defense against the infection (Dornand et al., 2002; Weiss and Schaible, 2015). Pattern recognition receptors (PRRs) such as Toll-like receptors (TLRs) on the phagocytes are considered to recognize *Bartonella* spp. (Kloch et al., 2018). Generally, LPS and particularly its lipid A part is mainly recognized by TLR4 and causes pro-inflammatory cytokines secretion to induce various inflammatory cells to move to the infection site (Malgorzata-Miller et al., 2016). It was reported that LPS of *B.henselae* and *B. bacilliformis* has a deep-rough structure, and *B.henselae* LPS contains an unusual lipid A with a long chain fatty acid and without an O-chain polysaccharide (Gorczynski et al., 2004; Focà et al., 2012). The unusual features of *Bartonella* LPS were weakly recognized by TLR4 and did not evoke TLR4 activation (Minnick, 1994; Raetz and Whitfield, 2002; Focà et al., 2012). As *B. henselae* LPS was 1,000-10,000-fold less active than *Salmonella* LPS in activating TLR4 signaling, *B. quintana* LPS could not induce pro-inflammatory cytokines production (Zähringer et al., 2004; Popa et al., 2007). And *B. quintana* LPS could be a TLR4 activation antagonist to inhibit release of cytokines mediated by *Escherichia coli* LPS, such as interleukin-1β, interleukin-6 and tumor necrosis factor α (Boonjakuakul et al., 2007; Popa et al., 2007; Matera et al., 2008). Moreover, it could also block TLR4 signaling transduction in rheumatoid arthritis (Abdollahi-Roodsaz et al., 2007). Compared with *Salmonella* flagellin, the flagellin of *Bartonella* species which possess flagella such as *B. bacilliformis, B. bovis, B. capreoli, B. chomelii, B. clarridgeiae*, and *B. schoenbuchensis* contains amino acid differences in the site of TLR5 recognition. Which does not cause flagellin-mediated TLR5-dependent NF-κB activation and might escape TLR5 recognition (Andersen-Nissen et al., 2005; Deng et al., 2012; Kloch et al., 2018). The unusual structures of LPS and flagellin are important for *Bartonella* spp. to escape the TLR4 and TLR5 recognition by phagocytes, respectively.

*Bartonella* adhesion A (BadA) is an outer membrane protein which is homologous to *Yersinia* adhesin A (YadA), *Haemophilus* surface fibrils (Hsf), *Moraxella* surface protein A (UspA), and *Haemophilus* adhesin (Hia) (Lafontaine et al., 2000; St Geme and Cutter, 2000; Biedzka-Sarek et al., 2008). BadA belongs to the trimeric autotransporter adhesion (TAA) family, which all share similar modular architectures, consisting of a head, neck/stalk repeats, and C-membrane anchor domains (Hoiczyk et al., 2000; Wollmann et al., 2006). The number of neck/stalk repeats are variable in different *Bartonella* species (Kaiser et al., 2012). BadA could cause bacterial autoaggregation and encode antigenic variation of repetitive tandem stalk domains to prevent phagocytosis (Riess et al., 2004). *Bartonella* could also temporarily enter macrophages in a unique *Bartonella*-containing vacuole (BCV) and delay lysosomal targeting and destruction (Kyme et al., 2005).

### The Third Strategy Is Preventing Complement Activation

The complement system has the function of microbial infection control, either directly by membrane-attack complex (MAC) formation or via phagocyte opsonization. It was considered that the absence of O-side chain of *Bartonella* LPS could decrease complement fixation and increase serum resistance (Zähringer et al., 2004). Recent observations suggested that BadA was involved in preventing complement activation, since mouse serum could kill *B. birtlesii* badA-knockout (ΔbadA) mutants, while not the wild type *B. birtlesii*. Moreover, anti-BadA antibodies could neutralized this killing activity and ΔbadA was resistant to heat-inactivated serum (Deng et al., 2012).

Since *Bartonella* LPS, flagellin, and BadA could inhibit the function of the immune system, such as the complement and phagocytic cells, the inflammatory response decreased resulting in reduced phagocytes migration, antigen presenting, and B cells activation.

## Step 2: Erythrocyte Adhesion

Intracellular pathogens must bind to host cells to successfully initiate infection (Barnett et al., 2015). Bacteria use various components to adhere to host cells, ranging from complex substances, such as fimbriae or pili, to proteins, such as *Brucella suis* BmaC, BtaE, and BatF adhesins (Ruiz-Ranwez et al., 2013; Wu et al., 2014). Recognition of host molecules by adhesins is the first step of bacterial infection (Coutte et al., 2003; Caswell et al., 2010; Ruiz-Ranwez et al., 2013). Exploitation of erythrocytes by *Bartonella* spp. is a complex progression through a series of different infection stages, beginning with erythrocyte adhesion. Although some factors have been shown to be essential for this step, the knowledge about erythrocyte adhesion factors of *Bartonella* is nominal. It is difficult to perform genetic studies, since no liquid medium can support rapid growth of *Bartonella* spp. and suitable animal models for study on pathogenicity of this bacteria are limited.

### The First Factor Is the Trw System

**Figure 2:**
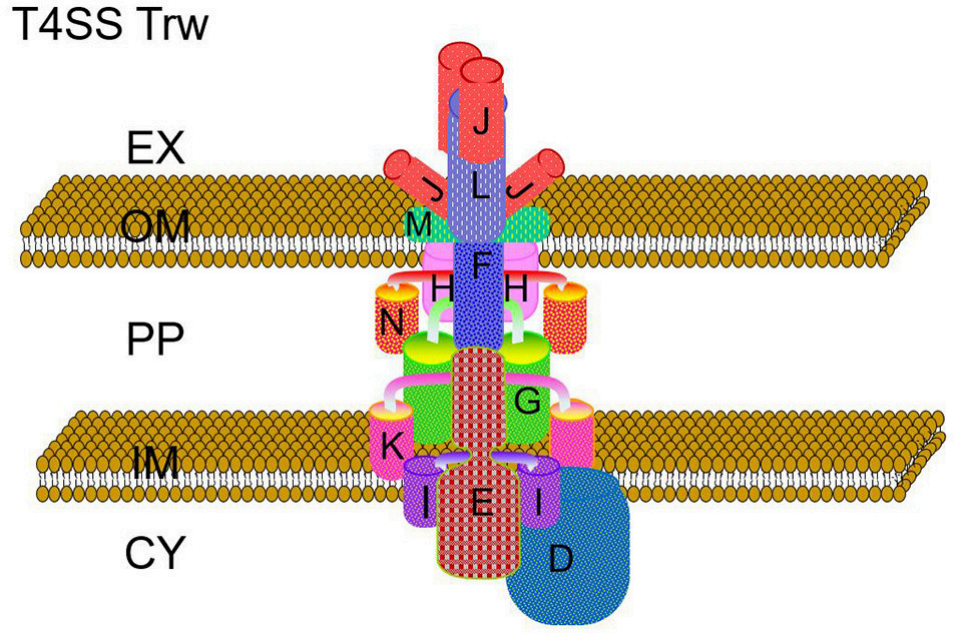
Hypothetical model of the architecture of the Trw system of *Bartonella* species. The Trw system is a multi-component protein complex spanning the inner and outer membranes of *Bartonella* species. The multiple copies of TrwL and TrwJ represent the major and minor pilus components, while the other duplicated proteins, TrwI and TrwH are involved in pilus elongation and for pilus anchorage to the outer membrane respectively. EX, extracellular matrix; OM, outer membrane; PP, periplasm; IM, inner membrane; CY, cytoplasm. Letters denote individual Trw subunits.

The Trw system is the third type 4 secretion system (T4SS) found in certain *Bartonella* spp. and has a short-path of evolution (Frank et al., 2005). It shares high homology with plasmid R388 which is a broad-host-range conjugation system of the IncW group that confers resistance to sulfonamide and trimethoprim and produces constitutively rigid conjugative pili called W pili (Bolland et al., 1990). Both encode an identical and interchangeable transcription regulatory circuit KorA/KorB repressor which could negatively regulate T4SS expression by binding to *kor* box sequences (Figure 2).

Although the Trw system shares homology with plasmid R388, this system lacks a TrwB which is the key protein required for transfering effectors. This suggests that the Trw system is not a secretion system (Seubert et al., 2003; Dehio, 2004; Vayssier-Taussat et al., 2010; Larrea et al., 2013). In fact, to date no substrates translocated by this system have been characterized. Upregulation of the Trw system during endothelial cell infection would decrease the time in the hostile environment of the bloodstream. *Bartonella* could then adhere to and invade erythrocytes quickly (Dehio, 2004; Pulliainen and Dehio, 2012). The Trw system required for intraerythrocytic infection was identified by large-scale signature tagged mutagenesis (STM) screens in the *B. birtlesii*-mouse and *B. tribocorum*-rat models *in vivo* (Saenz et al., 2007; Vayssier-Taussat et al., 2010).

Recently, the function of Trw in erythrocyte infection was identified by an *in vitro* model for erythrocyte adhesion and invasion (Vayssier-Taussat et al., 2010). In the study, we identified nine mutants that could not invade erythrocytes *in vitro*. The nine mutants included an invasion-associated locus (*ialA/B*) mutant, a leucine-isoleucine-valine (*livG*) mutant, and seven mutants for genes encoding Trw components. When we horizontally transferred the *trw* locus of rat-specific *B. tribocorum* into human-specific *B. quintana* and cat-specific *B. henselae*, they were able to interact with rat erythrocytes, suggesting that the Trw sytstem is a key factor of erythrocyte infection and host specificity.

**Figure 3:**
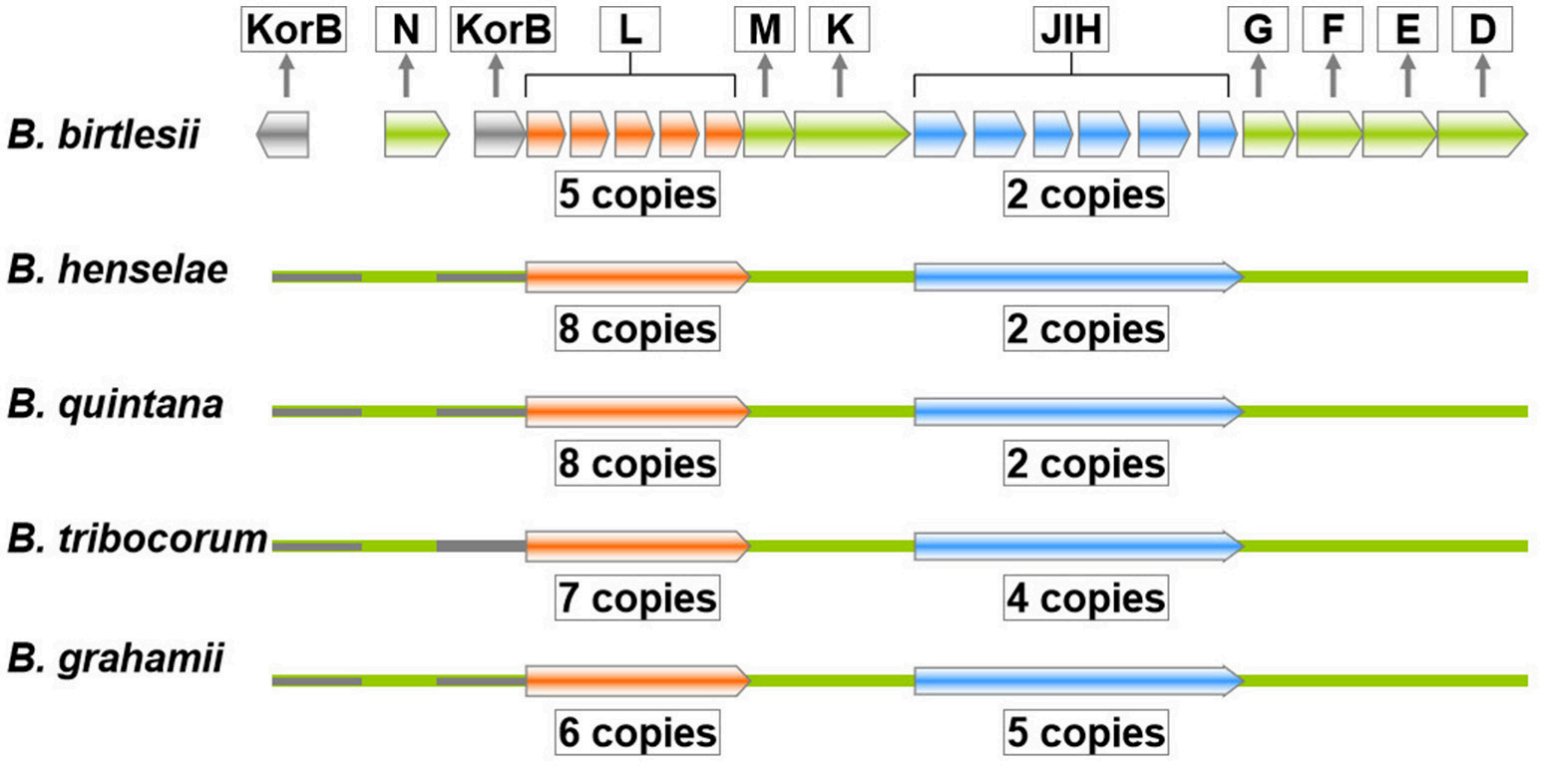
Gene order structure of the *trw* locus of *Bartonella* species. The *trw* genes of *Bartonella* species are collinear with the respective genes of plasmid R388, except for the multiple tandem gene duplications of *trwL* and *trwJ-I-H*, which are present in variable copy numbers in the different species. The copy number of amplified genes or segments of the *trw* locus of five *Bartonella* species is indicated within the boxes. The copy number *trwJ* and *trwL* displays a large degree of sequence variation.

**Figure 4:**
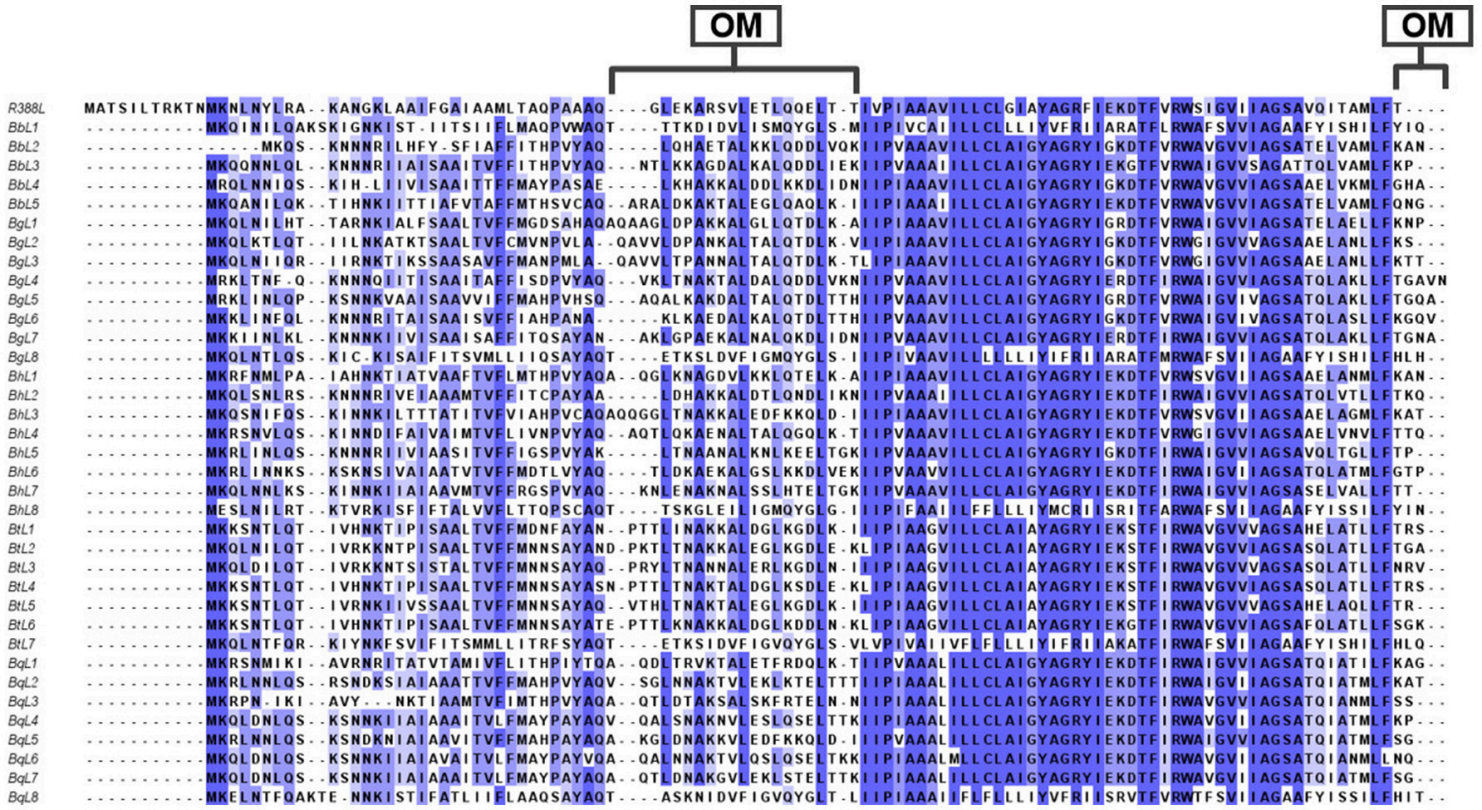
Multiple amino acid sequence alignments of TrwL of *Bartonella* species. Multiple amino acid sequences of TrwL from different *Bartonella* species of *B. birtlesii* (*BbL1* to *BbL5*)*, B. grahamii* (*BgL1* to *BbL7*), *B. henselae* (*BhL1* to *BhL8*), *B. quintana* (*BqL1* to *BhL8*), *B. tribocorum* (*BqL1* to *BhL7*), and R388 TrwL (*R388L*) were aligned by Clustal Omega. Conserved amino acids are shaded and each shade represented a degree of conservation (Blue, 100%). The major outer membrane proteins were calculated by TMHMM 2.0. All the sequences were taken from the NCBI GenBank. OM, Outer membrane proteins.

The *trw* genes of *Bartonella* species are collinear with their homologous genes of plasmid R388, except for the gene duplications of *trwJ-I-H* (the *virB5, virB6*, and *virB7* homologs) and *trwL* (the *virB2* homolog) (Figure 3). There are variable copy numbers of the duplicated genes in the different species, which is evidence of gene conversion and rapid evolution (Schröder and Dehio, 2005; Schulein et al., 2005; O'Rourke et al., 2011). For example, the trans and inner membrane regions of TrwL are almost identical, but the outer membrane regions are different across *Bartonella* species (Figure 4). The duplicated copies of *trwJ* and *trwL* encode variant forms of pilus components, while *trwH* and *trwI* are involved in pilus elongation and anchorage, respectively (Dehio, 2004; Deng et al., 2012).

**Figure 5:**
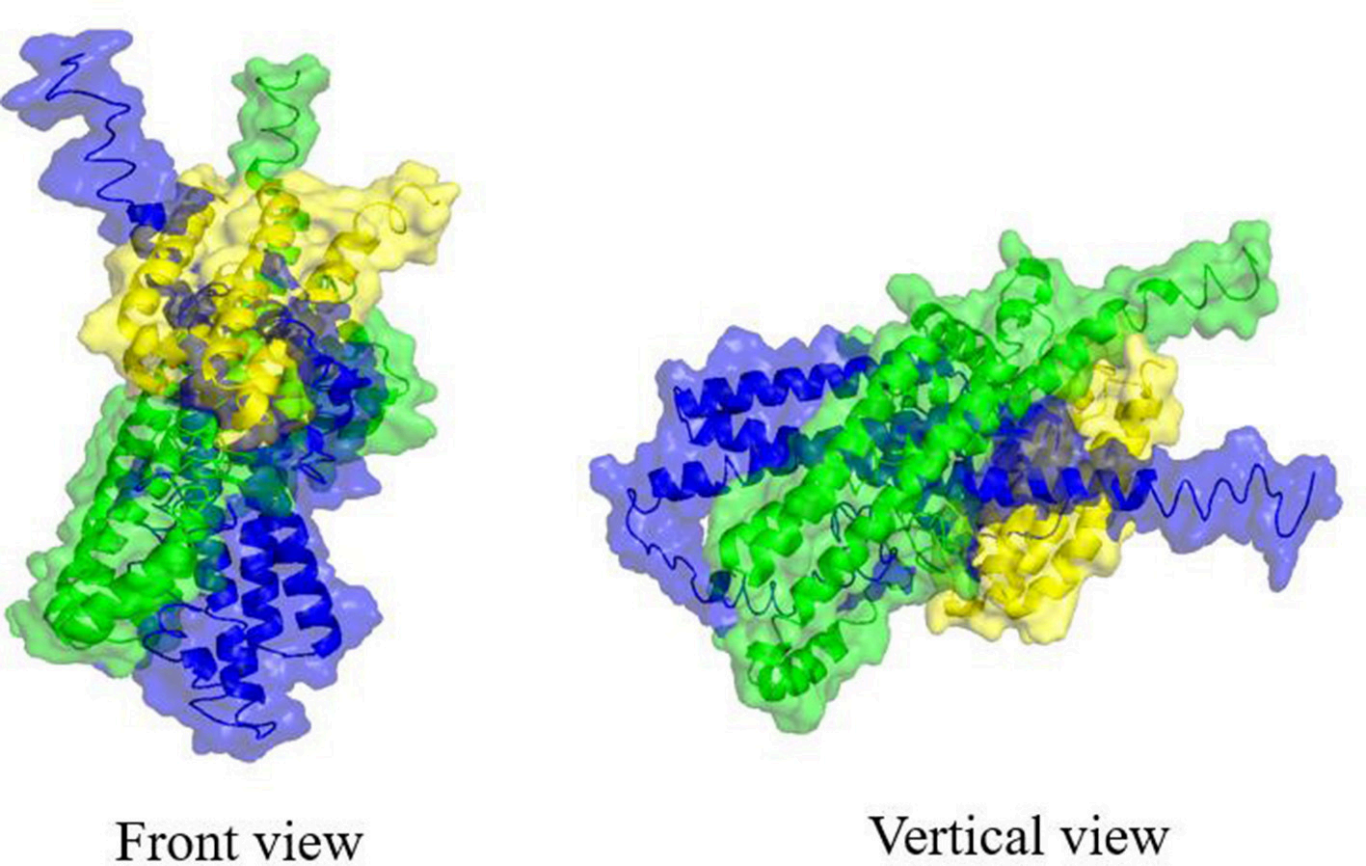
Molecular docking between *B. birtlesii* TrwJ1, TrwJ2, and TrwL. Three-dimensional models of *B. birtlesii* TrwJ1, TrwJ2, and the outer membrane parts of TrwL were modeled by the I-TASSER server based on the amino acid sequences (Yang et al., 2015). The best identified structural analogs of both TrwJ1 (C-score of −1.27 and TM-score of 0.74) and TrwJ2 (C-score of −1.45 and TM-score of 0.69) in protein data bank (PDB) are 1R8I, and the outer membrane parts of TrwL (C-score of −3.17 and TM-score of 0.70) in PDB is 4TQL. Molecular docking between *B. birtlesii* TrwJ1, TrwJ2 and the outer membrane parts of TrwL was analyzed using the ClusPro 2.0 server and visualized by the PyMOL program (Kozakov et al., 2017). The front and vertical view of the docked conformations were shown as *B. birtlesii* TrwJ1 (green cartoon with semitransparent surface), TrwJ2 (blue cartoon with semitransparent surface), and the outer membrane parts of TrwL (yellow cartoon with semitransparent surface).

However, the direct function of Trw system has only recently been obtained by using different technologies (Deng et al., 2012,a). In the study, both TrwJ1 and TrwJ2 were found at *B. birtlesii* surface and could bind to band 3 of mouse erythrocytes. It was considered that TrwL might also bind to the surface of erythrocytes, and the outer membrane parts of TrwL proteins might be responsible for this ability. Further studies are required to identify the erythrocytic receptors of TrwL. Bacteria usually use some virulence factors to bind to host cells more intimately after initial adherence. We considered that the specific and stable interactions between *Bartonella* and host erythrocytes were mediated by both TrwJ and TrwL. Moreover, TrwJ might have the capability to interact with TrwL, which represents the major and minor pilus (Figure 5). TrwJ and TrwL might be involved in initial or intimate adhesion during infection of erythrocytes. The interactions of TrwJ and TrwL with host erythrocytes were associated with *Bartonella* invasion, although direct evidence is lacking to confirm this theory.

The multiple gene copies have many advantages. First, they can increase expression of the dosage of pilus proteins and the length or the number of pili for rapid adhesion to erythrocytes in the bloodstream of the mammalian host (Gillespie et al., 2015). Second, multiple Trw pilus variants encoded by those gene copies might bind to various surface components of different blood groups in different reservoir populations (Dehio, 2008). Third, they provide variability within the genome for antigenic variation (Lindroos et al., 2006). Fourth, they might increase potential for new functions.

### The Second Factor Is Flagella

The role of Trw evolved to replace the flagella, since the expression of Trw and flagella is mutually exclusive among the *Bartonella* species (Dehio, 2008; Harms and Dehio, 2012). The multiple flagella which let *B.bacilliformis* with highly motile could be important for the high rate of erythrocyte infection in Oroya fever (Scherer et al., 1993; Dehio, 2001). It has been reported that *B. bacilliformis* flagellin site-directed mutants bind poorly to erythrocytes, and this phenomenon can be partially rescued by trans-complementation with nature flagellin (Battisti and Minnick, 1999; Sander et al., 2000). The flagellin subunit antibodies could partially inhibit the adhesion between *B. bacilliformis* and erythrocytes (Scherer et al., 1993; Sander et al., 2000). Early work indicated that *B. bacilliformis* could interact with many human erythrocyte membrane proteins, including glycophorins A and B (Buckles and McGinnis Hill, 2000). Those observations correspond with the former views that the flagella of *Bartonella* may serve as an adhesin, although it remains unknown whether flagella can directly bind to host erythrocytes (Walker and Winkler, 1981; Benson et al., 1986).

## Step 3: Erythrocyte Invasion

After erythrocyte adhesion, *Bartonella* invaded mature erythrocytes within 2 days, which has been demonstrated in the *B. tribocorum*-rat infection model (Seubert et al., 2002). The unusual structure and physiology of erythrocytes could allow *Bartonella* to escape antigen presentation and immune surveillance. We have little knowledge about how *Bartonella* enter host erythrocytes, but some factors have been shown to be essential for this step.

### The First Factor Is IalB

As described above, IalA/B was identified by STM screens in the *B.birtlesii*-mouse and *B. tribocorum*-rat models *in vivo* and by an *in vitro* model for erythrocyte adhesion and invasion.

**Figure 6:**
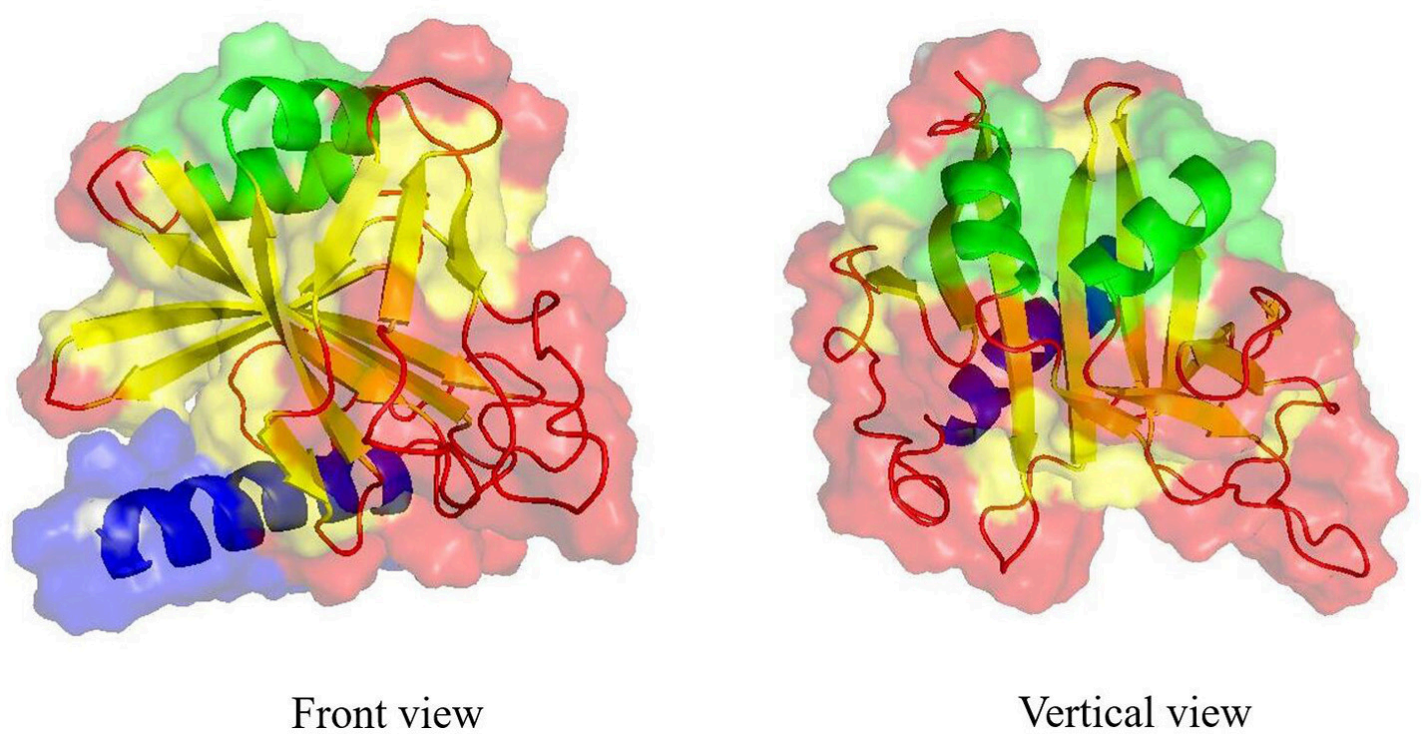
Three-dimensional structure model of *B. birtlesii* IalB. Three-dimensional structure model of *B. birtlesii* IalB was built using the I-TASSER server based on the amino acid sequence (Yang et al., 2015). The front and vertical view of the crystal structure of IalB (C-score of −1.15 and TM-score of 0.77) was shown as the cartoon model with semitransparent surface. The best identified structural analogs in PDB is 3DTD. The structure that contained 1 signal peptide domain (blue bands), 2 α-helices (green bands), 11 β-strands (yellow bands), and 14 coils (red bands) in the secondary structure constituted a stable region.

IalB which is a 19.9 kDa protein with putative signal peptides (Figure 6), shares high homology with the *Yersinia enterocolitica* protein Ail, that plays a major role in cell invasion (Kirjavainen et al., 2008; Deng et al., 2016). Early work demonstrated that *E. coli* could invade erythrocytes when it was transformed with *B. bacilliformis ialB*, and deletion of *ialB* decreased the erythrocyte infection of *B.birtlesii* and *B.tribocorum in vivo* (Mitchell and Minnick, 1995; Saenz et al., 2007; Vayssier-Taussat et al., 2010). Moreover, the *B. birtlesii* IalB mutant caused a 10-fold decrease in erythrocyte invasion, but it has no significant effect on erythrocyte adhesion *in vitro* (Vayssier-Taussat et al., 2010). The *B. bacilliformis* mutant can be restored to erythrocyte invasiveness when trans-complemented with wild-type IalB locus (Coleman and Minnick, 2001). Our recent study showed that IalB was immunogenic and anti-IalB antibodies could inhibit mouse erythrocyte invasion by *B. birtlesii* (Deng et al., 2016).

There was confusion about the location of IalB in *Bartonella. B. bacilliformis* IalB was an inner membrane protein, while *B. henselae* IalB was an outer membrane protein (Mitchell and Minnick, 1995; Coleman and Minnick, 2001; Chenoweth et al., 2004). In our recent study, a small quantity of IalB was detected on *B. birtlesii* surface, while most of IalB was expressed in *Bartonella* lysate supernatants of different species (Deng et al., 2016). So, we hypothesized that most of the *B. birtlesii* IalB might be secreted proteins that mediated erythrocyte invasion by unknown mechanisms.

### The Second Factor Is Deformin

*B. bacilliformis* could cause production of trenches, pits, conical invaginations, and internal vacuoles in the erythrocyte membrane (Benson et al., 1986; Xu et al., 1995). This phenomenon is mediated by deformin, which has been found in the culture supernatants of *B. henselae* and *B. bacilliformis*, suggesting that this mechanism might be present in several *Bartonella* species (Iwaki-Egawa and Ihler, 1997).

There was confusion about the identity of deformin in *Bartonella*. Early work demonstrated that deformin was a protease- and heat- resistant, water-soluble, and albumin binding molecule with a molecular weight of ~1.4 kDa (Derrick and Ihler, 2001). More recent work has indicated that deformin is several proteins present in the supernatant of *B. bacilliformis* with a molecular weight of ~36 kDa (Hendrix and Kiss, 2003). The 36 kDa proteins appear to either necessary for deformin secretion or directly deforming human erythrocytes. The nature of deformin and the molecular mechanisms of erythrocyte deformation require further studies.

### The Third Factor Is Hemolysin

Two types of *Bartonellla* hemolytic factors have been found including a contact-dependent hemolysin of *B. bacilliformis* and an autotransporter cohemolysin of *B. henselae* (Hendrix, 2000; Litwin and Johnson, 2005). *B. bacilliformis* contact-dependent hemolysin is maximally expressed during exponential growth phase, and might be used to escape from the vacuoles or erythrocytes during intracellular parasitism (Hendrix, 2000; Litwin and Johnson, 2005). *B. henselae* cohemolysin which is a 180 kDa autotransporter protein, has homologs in *B. quintana* and causes lysis of erythrocytes (Litwin and Johnson, 2005; Minnick and Battisti, 2009).

## Step 4: Erythrocyte Replication and Persistence

*Bartonella* spp. attach, invade and replicate within a vacuole of erythrocytes in the *B. tribocorum*-rat infection model. After several days, bacterial replication stops until an approximately eight daughter cells are reproduced. There are some subtle changes in the physiology of erythrocytes during erythrocyte invasion and replication. *B. tribocorum*-infected erythrocytes are removed more rapidly than uninfected erythrocytes from circulation. However, once the number of intraerythrocytic *Bartonella* reaches static levels, the distinguishable changes and the rapid clearance rates will disappear (Schülein et al., 2001). Within an erythrocyte, *Bartonella* must not only get nutrients, but also cope with stressors.

### The First Strategy Is Nutrient Uptake

*Bartonella* species use two gene families of heme binding proteins (Hbps) and the heme utilization locus (Hut) to sequester heme (Carroll et al., 2000; Minnick et al., 2003; Zimmermann et al., 2003; Parrow et al., 2009). Hbps are required for intraerythrocytic bacteraemia and have been identified by STM screens in the *B. birtlesii*-mouse and *B. tribocorum*-rat models *in vivo* (Saenz et al., 2007; Vayssier-Taussat et al., 2010). *B. quintana* HbpA is a 29.3 kDa protein and part of a *hbpA-E* gene family (Carroll et al., 2000). Compared with parental strains, an HbpA mutant of *B. quintana* showed an enhanced heme binding phenotype (Minnick et al., 2003). It was also reported that anti-HbpA antibodies could inhibit the hemin binding in a dose-dependent manner (Carroll et al., 2000).

LivF and LivG, which are highly conserved among the *Bartonella* species, are required for intraerythrocytic bacteraemia and have been identified by STM screens in the *B. birtlesii*-mouse and *B. tribocorum*-rat models *in vivo*. Moreover, the *B. birtlesii* LivG mutant provoked a dramatic decrease in bacterial entry into erythrocytes *in vitro* (Vayssier-Taussat et al., 2010). LivF and LivG which are ATPase components of ABC transporters are required for amino acid nutrient uptake during *Bartonella* inside erythrocytes (Saenz et al., 2007).

### The Second Strategy Is Against Stressors

In order to adapt to the intraerythrocytic environment, *Bartonella* must cope with a variety of stressors, including reactive oxygen species, fluctuations in osmolarity, changes in pH, and misfolded proteins.

IalA which is a 20.1 kDa protein, has homologs in other invasive bacteria and has been demonstrated as a (de)nucleoside polyphosphate hydrolase of the MutT motif family (Mitchell and Minnick, 1995, 1997; Cartwright et al., 1999; Conyers and Bessman, 1999). IalA hydrolysates including ATP and inorganic phosphate could he recycled. IalA and its homologs are believed to regulate the level of stress-induced nucleotides and their derivatives during invasion. The carboxy-terminal protease (CtpA), which is encoded upstream of the *ialA* gene, could degrade misfolded or aberrant proteins from stress or anomalous processing (Mitchell and Minnick, 1997; Cartwright et al., 1999).

It has been reported that BatR/BatS which is an important two-component regulator/sensor is probably used by *Bartonella* to regulate the expression of some pathogenic genes such as the T4SS, BadA, and Hbps, and respond to environmental cues in the mammalian circulatory system (Quebatte et al., 2010; Harms and Dehio, 2012).

None of the molecular factors of mechanisms allowing for *Bartonella* spp. replication and persistence in the infected erythrocytes have been identified to date.

## Conclusion

*Bartonella* species are intraerythrocytic pathogens. They are mainly transmitted by animal contact and arthropods. For example, *B. henselae* is transmitted between cats by cat fleas (*Ctenocephalides felis*) and transmitted from cats to humans by cat scratches or bites (Chomel et al., 1996). In order to prevent the spread of the disease, it is important for scientists to explore the mechanisms of *Bartonella* infection.

Despite significant amounts of effort and advances to understand the molecular mechanisms of how *Bartonella* infects host erythrocytes, many uncertain aspects need further studies. The functions of the above-mentioned strategies and virulence determinants are still not fully elucidated and many other virulence factors have yet to be found. Moreover, the gene expression, regulation, and signal transduction pathways of those factors are still elusive. We also know little about the physiological changes and recognition receptors of erythrocytes during their infection.

In summary, with so many exciting and important questions yet to be answered, future studies would not only better clarify the functions of the factors, but also increase our understanding of the network between the factors and erythrocytes at a molecular level.

## Author Contributions

HD, QP, and BZ wrote the initial draft of the paper. HD and MV-T organized and proofread the paper. BZ helped to draft the figure. HD approved the version to be published. All authors read and approved the final manuscript.

### Conflict of Interest Statement

The authors declare that the research was conducted in the absence of any commercial or financial relationships that could be construed as a potential conflict of interest.
